# Stress, student burnout and study engagement – a cross-sectional comparison of university students of different academic subjects

**DOI:** 10.1186/s40359-025-02602-6

**Published:** 2025-03-24

**Authors:** Nils Olson, Renate Oberhoffer-Fritz, Barbara Reiner, Thorsten Schulz

**Affiliations:** TUM School of Medicine and Health, Chair of Preventive Pediatrics, Technical University of Munich, Georg-Brauchle-Ring 60/62, 80992 Munich, Germany

**Keywords:** Stress, Student burnout, Study engagement, University students, Study fields

## Abstract

**Background:**

Stress and burnout among students are health concerns in higher education systems, the prevalence of which exceeds that of the working population. Both are associated with impaired health and increased university drop-out rates. Study engagement, a positive study-related attitude characterized by energy, dedication, and absorption, counteracts stress and burnout. Person-oriented approaches can help to ensure students’ health and well-being and help to deduce preventive measures and interventions. Nevertheless, most studies treat students as a homogenous group and do not differentiate between academic subjects. Students apart from medical and nursing sciences have been mostly neglected within this research field.

**Methods:**

In a cross-sectional study, a sample of *n* = 947 students from five academic subject fields (Informatics, Mechanical Engineering, Sports and Health Sciences, Medicine, Economic Sciences) at a university in Germany was analyzed using an online survey. Sociodemographic data, perceived stress, study engagement and student burnout were included.

**Results:**

A total of 73.2% of the students were moderately to highly stressed, with females displaying higher stress levels than males. Almost one-third of the students reported frequent symptoms of burnout, while 44.5% reported a high degree of study engagement, with no differences according to sex. Stress (male: F [4, 401] = 5.321; *p* < 0.001; female: F [4, 532] = 9.325; *p* < 0.001), burnout (F [4, 938] = 7.537–11.827; *p* < 0.001) and engagement (F [4, 938] = 14.426; *p* < 0.001) were significantly differentiated by faculty affiliation. Medical students had the lowest stress levels, while informatics students had the highest stress levels. The faculty of informatics also had the highest level of burnout symptoms, while medical students and students in sports and health sciences displayed the most beneficial values. Medical students were most engaged, differing significantly from those of all the other faculties.

**Conclusions:**

Stress and burnout seem to be a problem in all students, especially among students in informatics and engineering. The previous focus on medical students concerning study demands does not seem justified according to our results. Fostering a supportive environment is key for study engagement, health and well-being of students. The inclusion of further individual factors should be a future concern in order to find and promote strategies for a healthy education system.

## Background

Chronic stress and burnout have been studied in regard to workplace functioning and occupational health and well-being for several decades now [1, 2]. They can also cause severe adverse effects for workers, leading to a loss of productivity, quality of life and overall health; additionally, they can also affect the economy and public health [3].

Burnout was originally associated with healthcare occupations only [4] and was seen as the result of prolonged emotional strain from intense engagement with people in the work environment [5]. Today, it is recognized that burnout can affect individuals across all professional fields [6]. Only recently has burnout been observed and studied in university students, where the term student burnout has been established [5].

Student burnout is defined as a state of reduced capacity for experience with concomitant emotional and physical exhaustion as well as depersonalization, the feeling of reduced coping and cognitive slowdown [7]. It is characterized by a combination of emotional exhaustion (EE), cynicism (CY) and a feeling of reduced academic efficacy (RAE) [8].

Study engagement forms the positive antipode of student burnout. In fact, student burnout is often considered as an erosion of academic engagement [5]. For the purpose of this study, study engagement is defined in three dimensions: vigor (i.e., having high levels of energy while studying), dedication (i.e., perceiving one’s studies as important and meaningful), and absorption (i.e., being immersed in one’s studies). They contribute to a positive, rewarding, and fulfilling state of mind, a high energy level, and positive study-related emotions. High engagement is related to positive health outcomes [9].

Generally, students are at high risk of experiencing significant stress and burnout. This is related not only to the specific stage of life and various changes in the individual’s lifestyle but also to the specific demands of studying and the instability this period of life brings [10–12]. In addition to the transition from late adolescence to young adulthood, students experience major shifts within their social environment and social roles, a detachment from family and parental home, the pursuit of educational and occupational choices, changes in romantic status and often a double burden of academic and occupational liabilities [13]. Students’ life structures and health competencies often fail to align with the newfound autonomy and responsibilities that come with these transitions, frequently leading to maladaptive coping strategies [14]. In Europe, an aggravating factor has been the introduction of the Bologna reform and its implications for students [15, 16]. The prevalent mental health issues, stress, negative coping mechanisms [17] and even suicidal ideation [18, 19] observed among students reflect the intense challenges posed by major life transitions and the high demands of university and work life. Student burnout has been linked to lower self-esteem, high university dropout rates and increased suicidal ideation, but to a few other factors [19–21].

Research on burnout and engagement among university students is limited, with significant variation in methodologies across studies. Nevertheless, it can be concluded that the prevalence of burnout is high in the student population, with rates ranging from 12% to more than 70% [22–27]. Thus, exceeding even the rates of workers in the medical fields [78]. Among those students who did not experience burnout in the cited studies, many individuals were already at increased risk for its development [22–27]. With only a few studies investigating the prevalence of study engagement, it can be assumed that barely half of the students are highly engaged within their study courses [22, 28, 29].

Research on student burnout often uses the Study-Demands-Resources (SD-R) model [30]. The SD-R model is a simple, resilience-based model derived from the Job-Demands-Resources model [31] and describes the existence of risk factors (demands) and protective factors (resources) for the development of burnout and engagement in the study environment. Frequently identified resources include staff support, scope of action, health behavior, and psychological flexibility. While mental health impairments are consistent demands [28, 32–34], semester progression, side occupation and academic workload have produced mixed results [29, 35]. Yet, further research is needed to explore these variables in more diverse student populations and consider new factors.

There is inconsistent evidence for the role of gender in engagement and burnout. Some studies have identified female sex as a risk factor for higher rates of burnout [25, 26], but mostly no differences have been observed, which is also applicable to study engagement [29, 33, 36].

Only few studies exist and those lack consistency in regards to methods and the included protective and risk factors. The two major omissions in previous research include the lack of person-oriented approaches, which would allow the identification of subgroups, enabling the customization of measures to meet the distinctive needs of specific target groups.

Second, previous studies have focused almost exclusively on students in medical fields or nursing sciences and have broadly neglected other disciplines [27, 37, 38]. Comparative studies are very rare. The few studies exploring other academic fields have also indicated higher stress levels among students in those areas [29, 36, 39]. Nevertheless, findings from medical fields are often either generalized to all students, or students in medical fields are highlighted as a particular vulnerable group without neither being substantially empirically confirmed.

In general, study engagement in conjunction with student burnout is still an underexplored topic within the higher education systems. Only a few person-oriented studies exist in this field, and often, those studies are limited to medical students. Other majors have been broadly neglected thus far. Based on this, the goals of this paper are as follows:

1) to investigate the prevalence of stress, burnout and engagement, thereby differentiating between different representative majors at a technical university.

2) to compare the different study fields to determine whether they comprise different subgroups with respect to stress, burnout and engagement.

These findings are relevant for identifying subgroups to deduce, customize and distinguish certain measures in the context of student health management.

## Methods

This study is based on a cross-sectional online survey of the Technical University of Munich. Details concerning the survey design can be obtained from our previous publication [29]. For the purposes of this research, data on age, sex, faculty affiliation, number of semesters, Utrecht Work Engagement Scale for Students (UWES-S 9), Maslach Burnout Inventory Short Form for Students (MBI-SS), and Perceived Stress Scale (PSS-10) were analyzed.

Participants were fully informed about the study’s aims and provided written consent before participating. The survey yielded responses from 4,720 students, resulting in a response rate of 10.3%, with 54.8% being female (*n* = 2,588), 44.7% male (*n* = 2,108), and 0.5% nonbinary (*n* = 24).

The study included five faculties covering various subject groups. Five faculties (Table 1) were selected, representing the diverse subject fields taught at the Technical University of Munich while limiting the number of groups to minimize Type I errors in the statistical analyses. Only bachelor’s degree and state examination students who answered all relevant questions were included, to control for potential differences between undergraduate and graduate students, resulting in a final sample of 947 students—42.9% male (*n* = 406), 56.7% female (*n* = 537), and 0.4% nonbinary (*n* = 4). Faculty affiliation distribution is detailed in Table 1.

**Table 1:** Faculty affiliation

Faculty	male	female	diverse	*N*
Informatics	167 (76.3%)	49 (22.4%)	3 (1.4%)	219
Mechanical Engineering	94 (63.9%)	53 (36.1%)	0	147
Sports and Health Sciences	54 (18.2%)	242 (81.8%)	0	198
Medicine	51 (25.8%)	147 (73.7%)	1 (0.5%)	296
Economic Sciences	40 (46.0%)	47 (54.0%)	0	87
Total	406 (42.9%)	538 (56.7%)	4 (0.4%)	947

### Student burnout and study engagement

For this study, burnout symptoms were measured using the Maslach Burnout Inventory Short Form for Students (MBI-SS), which assesses three dimensions: emotional exhaustion (EE), which reflects fatigue due to study demands and represents the individual stress component; cynicism (CY), indicating a mental distancing from studies and detached responses to peers and teachers, representing the interpersonal component; and reduced academic efficacy (RAE), indicating a sense of decreased competence and productivity and a diminished sense of accomplishment, representing the self-evaluation component [4, 40, 41]. The MBI has been internationally validated for measuring student burnout [42, 43]. Categories were established, distinguishing between individuals who experienced ‘frequent’ symptoms, defined as at least once a week, and those with ‘infrequent’ symptoms, occurring less than once a week. Cronbach’s Alpha values were 0.831 for EE, 0.867 for CY, and 0.746 for RAE.

Study Engagement was measured using the Utrecht Work Engagement Scale Student Version (UWES-S 9), which evaluates engagement levels in students through three dimensions: vigor (high energy levels while studying), dedication (finding one’s studies important and meaningful), and absorption (deep immersion in one’s studies) [5]. This scale has international validation [5, 32, 44, 45].

The MBI and UWES-S have both been proven to maintain their validity, reliability and consistency in their respective German versions, which have been used in our study [43, 46]. The MBI and the UWES have been processed and analyzed, based on the methods in our previous study [29].

### Perceived stress Scale – PSS

Several instruments have been used to assess stress in college students. One instrument is the Perceived Stress Scale (PSS-10), which measures perceived stress and reactions to stressful situations. It has been correlated with several psychological and physiological scales. The PSS-10 measures self-reported stress and was used because of its established validity and reliability [47], which has been confirmed in its German version [48]; it includes 10 questions whose responses are rated on a 5-point Likert scale and assesses stressful experiences and responses to stress over the previous four weeks. Negative responses are reverse-scored to ensure correct interpretation to the effect that higher overall scores indicate higher stress levels. The sum of the scores ranges from 0 to 40 [49]. Reference scores for different age groups and sexes have been published and can be compared to the collected data [50]. Additionally, groups were assigned stress scores as follows: 0–13 for low stress, 14–26 for moderate stress, and 27 or higher for severe stress [46]. These categories are from arbitrary thresholds, that have been performed in the student population before [51, 52]. The internal reliability (Crohnbach’s α) was 0.892 in our sample.

### Statistical analysis

Descriptive data are reported as mean (M) ± standard deviation (SD) for metric variables and as frequencies and percentages for categorical variables. Pearson’s correlation analysis examined relationships among metric variables.

The high internal correlation among UWES-S 9 dimensions (Vigor, Dedication, Absorption (0.74–0.87) led to using a one-factor structure for this scale [53], while maintaining the three-factor structure for the MBI-SS due to the distinctiveness of the subscales. Differences between two groups were analyzed using Student’s t-test, with Cohen’s d indicating effect size. Analysis of variance (ANOVA) was used for comparing more than two groups, and Welch-ANOVA was employed when homogeneity was violated. Due to the high number of groups and the concomitant risk of type error I inflation, Tukey’s test has been chosen for pairwise comparison due to its robustness in that regards [54, 55]. For Welch-ANOVA Games Howell post-hoc comparison has been applied. Due to the small number of non-binary participants, they were excluded from sex-differentiated calculations but not from descriptive statistics.

Statistical significance was set at *p* ≤ 0.05. All analyses were performed using SPSS 29.0 (IBM Inc., Armonk, New York, USA).

## Results

Only those participants who were of legal age, were part of a bachelor’s degree or state examination program and who completed all of the relevant questions were included; thus, 947 students were included—42.9% male (*n* = 406), 56.7% female (*n* = 537) and 0.4% nonbinary (*n* = 4). Age ranged from 18 to 51 years and averaged 21.4 ± 3.06 years, with females (21.6 ± 3.39 years) being slightly but significantly older than males (21.2. ±2.56) (t(942)=-2.282; *p* = 0.018; *n* = 944).

The students were between the 1st and 16th semester of their current degree program, with a mean of 4.0 ± 2.89 semesters. A large proportion of participants were students in their first or second semester (*n* = 297, 31.6%). The third and fourth semesters accounted for 26.8% (*n* = 253), the fifth and sixth semesters accounted for 20.7% (*n* = 195), the seventh to ninth semesters accounted for 16.0% (*n* = 150) and the tenth and above semesters accounted for 4.9% (*n* = 46). The number of semesters already studied has a minor but significant influence on the constructs of stress, the dimension of RAE, and study engagement, all being negatively directed and having small effect sizes.

The students scored 18.3 ± 6.95 on average on the Perceived Stress Scale (PSS-10), with 26.8% of the students having low levels of stress per definition, 59.9% having moderate levels of stress and 13.3% having high levels of stress. Females scored significantly higher (19.7 ± 6.87) than males (16.4 ± 6.51) (t(942)=-7,395; *p* < 0.001; *n* = 944). Compared to an 18- to 29-year-old reference population (14.2 ± 6.2) [50], a significantly greater score on the PSS-10 was found among the students in this sample (t(1, 946) = 18.377; *p* < 0.001).

The mean score for burnout in the EE dimension was 2.6 ± 1.52. The mean CY score was 1.1 ± 1.36, and the mean RAE score was 2.0 ± 1.53. A total of 28.2% (*n* = 267) of the students had frequent symptoms in at least one dimension. Accordingly, 22.5% (*n* = 213) of the students frequently experienced symptoms of EE, 6.0% (*n* = 57) symptoms of CY and 13.6% (*n* = 129) had frequent symptoms of RAE. A total of 3.0% (*n* = 28) had frequent symptoms in all three dimensions simultaneously. There was no difference between males and females in any of the burnout dimensions. A total of 6.1% (*n* = 58) of the students were simultaneously engaged and burned out in at least one dimension.

With respect to study engagement, the average score on the UWES-S-9 was 3.4 ± 1.04, with no significant difference between males and females. The majority of the students (55.5%, *n* = 526) were categorized as having low to medium study engagement, whereas 44.5% (*n* = 421) were highly engaged.

The correlation analysis revealed strong interrelations among the three burnout dimensions (Table 2). Study engagement had a medium to strong negative correlation with all three burnout dimensions, with CY being associated to the greatest degree. Perceived stress is moderately to strongly associated with all three burnout dimensions and moderately negatively associated with study engagement.

**Table 2:** Means, standard deviation and correlation with correlation coefficient [r]

Variable	Mean	SD	1	2	3	4	5	6
1. Emotional Exhaustion	2.6	1.52						
2. Cynicism	1.1	1.36	0.48**					
3. Reduced academic efficacy	2.0	1.53	0.59**	0.54**				
4. Study Engagement	3.4	1.04	− 0.40**	− 0.62**	− 0.46**			
5. Perceived Stress	18.3	6.95	0.58**	0.40**	0.58**	− 0.38**		
6. Age [years]	21.4	3.06	− 0.08*	− 0.01	− 0.07*	− 0.02	− 0.04	
7. Semester	4.0	2.89	− 0.15**	0.05	− 0.14**	− 0.02	− 0.14**	0.50**

*significant at the level of *p* ≤ 0.05; ** significant at the level of *p* ≤ 0.001

One-factor analyses of variance (ANOVA) were performed to identify group differences between the faculties in regard to stress, burnout and study engagement. For stress, the sample was divided into male and female participants because the PSS-10-mean differed significantly between these two groups. The Faculty of Information Technology had the highest stress score among the male students, and the Faculty of Medicine had the lowest stress score (Table 3). According to the ANOVA, the faculties differed significantly in regard to male stress levels (F(4, 401) = 5.321; *p* < 0.001), with the students of Information Technology differing significantly from those of Medicine, with an effect size of d = 0.575 and Economic Sciences (d = 0.467) in the post-hoc test.

Among the female students, the Faculty of Information Technology had the highest score on the PSS-10, while the students of Medicine displayed the lowest score. The differences between the faculties were significant according to the Welch ANOVA (F(4, 532) = 9.325; *p* < 0.001), with the Faculty of Medicine having significantly lower values than all the other faculties in post-hoc testing. The between-faculty contrasts showed a large effect size (d = 0.814) when comparing Medicine to Information Technology, medium effect sizes for comparisons of Medicine to Engineering (d = 0.539), Medicine to Economics (d = 0.588) and Sports and Health Sciences to Information Technology (d = 0.654) [56].

**Table 3:** Group differences between faculties; ANOVA and pairwise comparison results; the highest and lowest values are highlighted

	Information Technology (I)	Engineering (E)	Medicine (M)	Sport and Health Sciences (S)	Economic Sciences (ES)	F	*p*	Eta^2^	Pairwise Tukey comparison
	MW ± SD	MW ± SD	MW ± SD	MW ± SD	MW ± SD				
Stress Male	**18.0 ± 7.16**	16.4 ± 5.52	**14.0 ± 5.83**	15.4 ± 5.67	14.7 ± 6.33	5.321	< 0.001	0.050	I > M; I > ES
Stress Female (Welch)	**23.5 ± 6.64**	21.4 ± 5.84	**17.9 ± 6.95**	19.2 ± 6.45	22.1 ± 7.77	9.325	< 0.001	0.066	I > M; I > S; M < E; M > ES

With respect to burnout, the Faculty of Information Technology scored the highest in the dimension of EE, while students affiliated with the Faculty of Sport and Health Sciences scored the lowest. The differences between the faculties were significant (F(4, 938) = 9.117; *p* < 0.001), with the students of Information Technology having significantly higher scores than those of Medicine (d = 0.424) and those of Sports and Health Sciences (d = 0.506). Students in the Engineering domain had significantly greater scores than students in the Sports and Health Sciences did (d = 0.324). For CY, Information Technology scored highest, and Medicine scored lowest, with a significant difference between the faculties according to the Welch ANOVA (F(4, 938) = 7.537; *p* < 0.001). The medical students had lower scores than did the students of all the other faculties according to the post-hoc test. The differences were of small to medium effect sizes (Information Technology: d = 0.499; Engineering: d = 0.472; Sports and Health Sciences: d = 0.334; Economic Sciences: d = 0.438). For the RAE, Information Technology and Engineering had the highest scores, and Medicine and Sports and Health Sciences had the lowest scores. Differences between faculties were significant (F(4, 938) = 11.827; *p* < 0.001) (Table 4). Pairwise testing showed that students in the medical field differed from those in the Information Technology (d = 0.459) and Engineering field (d = 0.448). Sport and Health Sciences differed significantly from Information Technology (d = 0.493), Engineering (d = 0.482) and Economic Sciences (d = 0.395).

With respect to study engagement, the Faculty of Medicine had the highest mean score, and Economic Sciences had the lowest. ANOVA tested positive for differences between faculties (F(4, 938) = 14.426; *p* < 0.001), with medical students differing from all other study programs in the post-hoc test (Table 4), with medium to large effect sizes (Information Technology: d = 0.584; Engineering (d = 0.593), Sport and Health Sciences: d = 0.524 and Economic Sciences; d = 0.738).

**Table 4:** Group differences between faculties; ANOVA and pairwise comparison results; the highest and lowest values are highlighted

	Information Technology (I)	Engineering (E)	Medicine (M)	Sport and Health Sciences (S)	Economic Sciences (ES)	F	*p*	Eta^2^	Pairwise Tukey comparison
	MW ± SD	MW ± SD	MW ± SD	MW ± SD	MW ± SD				
Exhaustion (Welch)	**3.1 ± 1.61**	2.8 ± 1.43	2.4 ± 1.56	**2.3 ± 1.36**	2.6 ± 1.57	9.117	< 0.001	0.061	I > M; I > S, E > S
Cynicism (Welch)	**1.3 ± 1.59**	1.2 ± 1.48	**0.6 ± 1.05**	1.0 ± 1.27	1.1 ± 1.24	7.537	< 0.001	0.052	M < I; M < E; M < S; M < ES
Reduced academic efficiency (Welch)	**2.3 ± 1.71**	**2.3 ± 1.56**	**1.6 ± 1.41**	**1.6 ± 1.31**	2.2 ± 1.54	11.827	< 0.001	0.074	M < I; M < E; S < I; S < M; S < ES
Study Engagement	3.2 ± 1.09	3.2 ± 1.05	**3.8 ± 1.01**	3.3 ± 0.93	**3.1 ± 0.87**	14.426	< 0.001	0.058	M > I; M > E; M > S; M > ES

## Discussion

The aim of the study was to identify the prevalence of stress, student burnout and study engagement among undergraduate students (bachelors and state examination programs) in different faculties at a German university. Differences between the faculties were examined, while sociodemographic factors were taken into account.

In comparison to a general reference population, the perceived stress level was significantly greater among the students in this study [50]. Only 26.8% of the students had a ‘low’ subjective stress level, while 13.3% of the students in the sample experienced ‘high’ stress levels according to the PSS. Women had significantly greater stress levels than men did in the total sample but also within each of the investigated fields of study. This sex difference is in line with previous findings [49, 57, 58]. Suggested causes for these sex differences are generally greater prevailing anxiety, less satisfaction with leisure time and more intensive and more frequent assessment and rumination of stressful situations among women [58]. Another reason is that the experience of stress is simply different for men and women, in the sense that women are more likely to internalize stress, while men are more likely to externalize it in the form of aggression and impulsivity [59].

For the three-factorial analysis of burnout, a distinction was made between students who scored an average mean of 4 or higher in each dimension and thus had frequent symptoms, defined as once or more per week, and those who showed symptoms less frequently. By that definition, 28.2% of the students reported frequent burnout symptoms in at least one dimension of student burnout. A total of 22.5% of the students experienced frequent symptoms of EE, 6.0% of CY and 13.6% of RAE. The number of students with EE is comparable to other populations in frequency and mean [60], while RAE is relatively high in this population compared to that in a German-wide investigation, which revealed that 24.4% of students were affected by frequent EE and that only 3.1% were affected by frequent RAE [22]. The number of students affected by CY, on the other hand, is less than that in the respective study, where up to 22.9% of the students were affected. However, the study population differed slightly in age, study field and male-to-female ratio.

In almost all of the cases where any frequent burnout symptom was present, frequent EE was also present and almost half of the respective students showed symptoms exclusively in EE. This may be attributed to the fact that EE is described as the initial burnout symptom, naturally occurring first and, with increasing persistence, potentially leading to additional symptoms in the other dimensions [61, 62]. Longitudinal studies are needed to confirm whether this progression is also true for student burnout. There was no significant difference in any of the burnout dimensions between male and female students, and also age had no relevant effect on these parameters.

In a previous study [29], we included students of all majors, including those within their master’s degree. We found slightly higher prevalence rates of burnout, which indicates that burnout symptoms could be more prevalent within the master’s program than within the bachelor’s programs.

44.5% of the study population were highly engaged. This is comparable to slightly less than has been previously shown in a German-wide investigation in 2017, in which 47.5% of the students displayed high study engagement [22]. Within our analysis, we did not find any differences between men and women. This finding adds to the body of evidence for both burnout and study engagement, which has to this day produced inconsistent findings regarding the role of sex. Despite the theoretical assumption that burnout is the consequence of depleted engagement [5], we found a small but substantial number of students (6.1%) who are simultaneously highly engaged but also show signs of frequent burnout-symptoms. This raises the question of how accurate the underlying framework of the relationship between burnout and engagement truly is. In addition, Loscalzo and Giannini [63] created the term studyholism, which is an excessive, compulsive focus on learning, driven by an overwhelming urge, which can lead to negative outcomes like stress, exhaustion, or neglect of other life areas. Being a novel construct, the relationship and distinctions to and from study engagement are not sufficiently explored, but it might be an important topic in regards to this finding. However, it is important to consider that our results are based on subclinical self-assessments.

The actual comparison of burnout and engagement between the different majors was performed using several analyses of variance. Regardless of sex, theFaculty of Medicine had the lowest stress levels among the included majors, and the Faculty of Information Technology had the highest stress level. This finding is quite remarkable, as medical students are often described as a very vulnerable group in regard to stress, with a particularly tight schedule and high demands [23]. We cannot provide data about objective workload and academic demands, but these results show that medical students deal with less subjective stress than do their fellow students in other disciplines. While females exhibit elevated stress levels compared to the general population, male medical students are the only student group within our sample who show comparable levels on average [50].

For the male students, the Faculty of Information Technology showed significantly greater PSS-10 scores than did the medical students and students of Economic Sciences. Female medical students scored significantly lower than did Information Technology, Engineering and Economic Sciences students, while Sports and Health Sciences students could also be differentiated from Information Technology students in the post-hoc test.

The faculty affiliation is significantly associated to the magnitude of burnout in all three dimensions. It becomes evident that students from the Faculty of Sports and Health Sciences and from the Faculty of Medicine expressed the most beneficial values in each dimension, whereas the students of the Faculty of Information Technology and from the Faculty of Engineering showed the least beneficial values. For EE, students in the Information Technology domain differed significantly from the students in the Medicine and Sports and Health Sciences domain. Moreover, engineering students are also significantly more affected by burnout symptoms than students in Sports and Health Sciences are. Post-hoc analysis revealed that medical students had significantly lower values for CY than did all other students. The same trend is observed when examining the RAE.

Post-hoc analyses revealed that students of Medicine had the highest values of study engagement; therefore, these students differed from all the other students from other faculties. This is not surprising given that medical students expressed the least amount of burnout symptoms in our sample because there seems to be an inverse relationship between burnout and engagement. Burnout is sometimes even depicted as a result of the erosion of engagement [5].

In former studies, mostly medical students were the subject of stress and mental health analyses. It was postulated that this group in particular is exposed to special demands [58]. According to the study-demands-resources model, this would lead to high levels of burnout and low levels of engagement, which we do not see in the present investigation. However, this study showed that the stress levels of female medical students are significantly greater than those of the general population and that all students, including those from the Faculty of Medicine, exhibit worrying burnout values. On the other hand, male medical students were the only group of students whose stress levels were descriptively on level with the reference population. Interestingly, the study showed that, relative to other student groups, medical students, regardless of sex, had the lowest stress levels. Compared to the Faculty of Information Technology, these differences were significant for both men and women.

In total, the students of the Faculty for Medicine and the Faculty of Sports and Health Sciences displayed the healthiest values in regard to stress, burnout and engagement in our study. Bringing these results in line with the current literature, it became quite startling that the claims that tout medical students as a very vulnerable student group are scientifically not reliable. In fact, only a few studies have investigated the differences between medical students and students in other disciplines. These studies, however, do show an ambiguous picture: Seedhom et al. [64] reported higher stress levels among Egyptian medical students than non-medical students, but the methodology has not been described sufficiently; e.g., there was no indication of sex distribution in the two samples. A study in Saudi Arabia [65] showed higher stress levels in medical students. A Turkish study also revealed poorer general health and mental health parameters among medical students [66]. On the other hand, El Gilany et al. [67] observed higher stress levels among law students than among medical students in an Egyptian sample. Mirza et al. [68] found no difference between medical and nonmedical students from Saudi Arabia in regard to stress and depression but found greater anxiety among the nonmedical students. On top of the ambiguous nature of these results, the applicability of these studies to Western universities is questionable. In older studies from the US [69] and Canada [70], higher stress levels were found among law students compared to medical students. A more recent study from Sweden [71] showed that business students had poorer mental health than medical students. A meta-analysis from 2016 could not identify significant differences in regard to depression between medical and nonmedical students [72]. Accordingly, the postulated vulnerability that we often see in the scientific literature does not seem to be based on evidence. On the contrary: the stress that has been noted in medical students may be a trend that is present among most university students [73] but rather attenuated among medical students. In initial burnout research, burnout was found to occur only as a result of prolonged emotional strain from intense engagement with people in the work environment. In that outdated definition, the interest in burnout among students would be more relevant for medical fields, but in fact, there are no comparative data with engineering or information technology occupations to our knowledge so far.

The subsequent question that needs to be addressed in future research is whether the differences between faculties are causal and if yes whether they are structural or personal. For example, Dahlin et al. [74] and Enns et al. [75] argued that medical students carry certain distinct personality traits, such as perfectionism and performance-based self-esteem. Dahlin et al. also concluded that the superior values of medical students in regard to stress could be attributed to the more cohesive structure of medical school and a greater awareness of a healthy lifestyle [71]. The latter would also be applicable for the students of Sports and Health Sciences and a reason for their better results in regards to stress, burnout and engagement. Furthermore, in Germany, very good final grades are required for the admission to medical school. It is therefore conceivable that most medical students are very proficient in academic learning. The requirements for the study courses at the other faculties are not as strict in regards to the numerus clausus and will result in a greater variance of final grades from school. In addition, mathematical and nature sciences study courses have a bad record of performing weeding-out courses during the first semesters, resulting in significant stress within the first exam phases.

Our findings show that all students exhibit an alarming mental health status, but within this group of students, medical and sports and health students have the most beneficial values concerning stress and burnout. However, the causality still needs to be analysed by future longitudinal and experimental study designs.

## Conclusions

There is a high prevalence of distress and burnout in the higher education setting, with two-thirds experiencing medium to high levels of stress and almost one-third being affected by frequent burnout symptoms. Less than half of the students were highly engaged. While women experience more stress, there are no gender differences in engagement or burnout. We found that stress, burnout and engagement are associated with faculty affiliation. Engineering and Information Technology students are prone to greater stress, more frequent burnout symptoms and less engagement, while students of Sports and Health Sciences and Medicine are the healthiest in this regard. How personal traits and what structural requirements contribute to the study demand resource model need to be further studied to clarify these findings.

The causal relationship cannot be assessed within our research approach, and the reasons for differences between faculties remain unclear. It seems possible that the demands and/or study satisfaction between faculties are different as these factors contribute to burnout and engagement to a great degree [29, 76, 77]. It is also possible that job prospects play a role or that certain fields attract students with certain characteristics that make them less or more resilient.

## Limitations

The study made a direct comparison of multiple subject fields in regards to stress, burnout and engagement among university students. The results are limited to the study design. For burnout, the presented results have to be taken in light of being a subclinical self-assessment and not a clinical diagnosis. In addition, the cross-sectional study design cannot give causal or temporal relationships. Longitudinal or experimental studies are needed to confirm causal relationships. The chosen target group were undergraduate students and therefore conclusions cannot be drawn towards other student groups, e.g., post-graduate study programs or even other universities.

Furthermore, the response rate of approximately 10% needs to be taken into account when interpreting and – especially – generalizing the results of the study. It is possible that more students with increased concerns or a poorer mental health have taken part in the questionnaire hereby skewing the results into the respective direction.

## Implication

Educators and institutions should learn about student burnout and engagement due to the high prevalence among students. Universities must help students recognize early burnout signs and offer support. While eliminating stressors is not feasible, fostering a supportive environment with stress management courses can improve student well-being and engagement.

The study found that all students face an increased risk of burnout, not just those in medical fields, as previously suggested. In contrast, more attention needs to be given to the other subject fields. These differences and differences between genders show that a one-size-fits-all approach to mental health and well-being cannot be effective.

Reasons for differences need to be further assessed and possible resources need to be identified and established as best-practice examples in order to strengthen university conditions in regards to well-being across faculties and universities.

However, further studies are needed to gain insights into the dependencies between study fields, study-related demands, lifestyle habits and health competences. This approach will help to better understand and individualize health promotion measures in the sense of behavioral and situational prevention at universities.

Future research should integrate structural, habitual, study-related, and personal factors into the SDR model to pinpoint key influences on student mental health. This will help tailor health promotion efforts, enhancing both behavioral and situational prevention at universities.

## Data Availability

The datasets used and analyzed in the current study are available in a highly anonymized form from the corresponding author upon reasonable request.

## Abbreviations

EE: Emotional Exhaustion
CY: Cynicism
RAE: Reduced Academic Efficacy
SD-R: Study Demands Resources Model
M: Mean
SD: Standard deviation
ANOVA: Analysis of Variences
PSS-10: Perceived Stress Scale 10
UWES-S: Utrecht Work Engagement Scale Student Version
MBI-SS: Maslach Burnout Inventory – Student Short Form
